# Pooling data from different populations: should there be regional differences in cerebral haemodynamics?

**DOI:** 10.1186/s12883-018-1155-8

**Published:** 2018-09-27

**Authors:** Angela S. M. Salinet, Ronney B. Panerai, Juliana Caldas, Ricardo C. Nogueira, Adriana B. Conforto, Manoel J. Texeira, Edson Bor-Seng-Shu, Thompson G. Robinson

**Affiliations:** 1 Neurology Department, School of Medicine, University of São Paulo, São Paulo, SP Brazil; 2 Biomedical Engineering, Engineering, Modelling and Applied Social Sciences Centre, Federal ABC University, Sao Bernardo do Campo, Sao Paulo, Brazil; 3 Faculty of Physiotherapy, Ibirapuera University, São Paulo, Brazil; 4 Department of Cardiovascular Sciences, Cerebral Haemodynamics in Ageing and Stroke Medicine Research Group, University of Leicester, Leicester, UK; 5 NIHR Leicester Biomedical Research Centre, University of Leicester, Leicester, UK; 6 Critical Care Unit Hospital São Rafael, Salvador, Brazil

**Keywords:** Cerebral haemodynamics, Acute stroke, Ischaemic stroke, Cerebral autoregulation, Transcranial Doppler ultrasound

## Abstract

**Background:**

Though genetic and environmental determinants of systemic haemodynamic have been reported, surprisingly little is known about their influences on cerebral haemodynamics. We assessed the potential geographical effect on cerebral haemodynamics by comparing the individual differences in cerebral blood flow velocity (CBFv), vasomotor tone (critical closing pressure- CrCP), vascular bed resistance (resistance-area product- RAP) and cerebral autoregulation (CA) mechanism on healthy subjects and acute ischaemic stroke (AIS) patients from two countries.

**Methods:**

Participants were pooled from databases in Leicester, United Kingdom (LEI) and São Paulo, Brazil (SP) research centres. Stroke patients admitted within 48 h of ischaemic stroke onset, as well as age- and sex-matched controls were enrolled. Beat-to-beat blood pressure (BP) and bilateral mean CBFv were recorded during 5 min baseline. CrCP and RAP were calculated. CA was quantified using transfer function analysis (TFA) of spontaneous oscillations in arterial BP and mean CBFv, and the derived autoregulatory index (ARI).

**Results:**

A total of 100 participants (50 LEI and 50 SP) were recruited. No geographical differences were found. Both LEI and SP AIS participants showed lower values of CA compared to controls. Moreover, the affected hemisphere presented lower resting CBFv and higher RAP compared to the unaffected hemisphere in both populations.

**Conclusions:**

Impairments of cerebral haemodynamics, demonstrated by several key parameters, was observed following AIS compared to controls irrespective of geographical region. These initial results should encourage further research on cerebral haemodynamic research with larger cohorts combining different populations.

## Background

Cerebral haemodynamic abnormalities may be useful early clinical markers in the development of age-related neurodegenerative disorders with vascular aetiologies [1], in determining an increased risk of stroke among healthy and diseased populations [2], and in predicting stroke prognosis [3, 4]. Due to its clinical relevance, in recent years, polling cerebral haemodynamic data from different international research centres has become, an attractive option to generate larger study cohorts, enhancing the statistical power and the generalizability of study results [5, 6]. Understanding to what extent regional differences affect cerebrovascular function is a key to planning international collaborative research and also to improve understanding of regional differences in treatment effectiveness and worldwide patient outcomes.

Health has multi-factorial determinants, including social, cultural, economic and environmental factors, genetic variation, and the quality of healthcare available [5]. Different continents have different health conditions, especially considering marked socioeconomic, ethnical and health care disparities. Moreover, recent studies have reported significant differences between continents in the prevalence of cardiovascular risk factors, such as diabetes mellitus [7, 8], hypertension [9], smoking [10] and hyperlipidaemia [11]. Nevertheless, little information on the possible international regional differences in cerebral haemodynamics is available.

Therefore, with the aim of pooling individual subjects data from different regions in an on-going international collaborative research study of cerebral haemodynamic changes following acute ischaemic stroke (AIS), we investigated systemic and cerebral haemodynamic parameters in healthy older and AIS populations derived from two different regional research centre databases in the United Kingdom and Brazil. Data from these two regions were chosen in order to ensure the homogeneity of the measurement settings and reduce the interference of other confounding factors (e.g. transcranial Doppler inter-operator reliability), as only one researcher (AS) collected and analysed the data.

## Methods

### Participants

Participants recruited by the same researcher (AS) using a standardized data collection and analysis protocol were included; participants from Leicester, United Kingdom (LEI) [12, 13] and Sao Paulo, Brazil (SP) were investigated. Stroke participants were consecutively recruited either from the Hyperacute Stroke Unit at the University Hospitals of Leicester NHS Trust (LEI) or from the emergency room at the Hospital das Clinicas of São Paulo (SP). Inclusion criteria were acute anterior circulation ischaemic stroke (< 48 h from stroke onset) confirmed by a single acute ischaemic brain lesion on magnetic resonance image (MRI) and/or computerized tomography (CT). Exclusion criteria comprised: (1) presence of intracranial or subarachnoid haemorrhage on CT or MRI; (2) significant ipsilateral carotid artery disease (> 50% stenosis); (3) absence of sufficient bilateral temporal bone window for insonation of the middle cerebral artery (MCA); (4) history of myocardial infarction within 6 months or other neurological disorder. Neurological impairment was assessed with the NIH Stroke Scale (NIHSS) on patient admission [14]. Age- and sex-matched healthy control subjects with no history of stroke or transient ischaemic attack, diabetes mellitus, hypercholesterolemia or carotid artery stenosis were also recruited from departmental staff and their relatives. All participants provided written informed consent in accordance with the Declaration of Helsinki; the Nottingham Research Ethics Committee 1 (LEI) and Ethics Committee of the Hospital das Clinicas (SP) approved the studies.

### Research protocol

As previously described [12, 13], measurements were performed with subjects in the supine position with slight elevation of the upper body. CBF velocity (CBFv) was measured in both MCAs by insonation through the temporal bone window with 2-MHz transducers attached to a head frame using a transcranial Doppler ultrasound (TCD) (Viasys Companion III; Viasys Healthcare and Doppler box, DWL for LEI and SP databases, respectively). Continuous non-invasive beat-to-beat blood pressure (BP) recording was performed with arterial volume clamping of the digital artery (Ohmeda 2300; Finapres, Louisville, CO, USA) with the subject’s hand (left for control and unaffected hand-side for stroke group) positioned at heart level. End-tidal CO_2_ (EtCO_2_) was monitored using an infrared capnograph (Capnocheck Plus and MX-200 and Transmai for LEI and SP database, respectively) during nasal expiration. After stable values had been established, the servomechanism of the Finapres device was turned off and a data segment of 5 min baseline was recorded to assess systemic and cerebral haemodynamic parameters.

Data were simultaneously recorded onto a data acquisition system (PHYSIDAS, Department of Medical Physics, University Hospitals of Leicester and Doppler Box, DWL for LEI and SP databases, respectively) for subsequent off-line analysis.

### Data analysis

All signals were visually inspected and narrow artefacts (< 100 ms) were removed by linear interpolation. CBFv channels were subjected to a median filter and all signals were low-pass filtered with a cut-off frequency of 20 Hz. The R–R interval was then automatically marked from the ECG and continuous heart rate (HR) plotted against time. Mean BP and CBFv values were calculated for each cardiac cycle. The end of each expiratory phase was detected in the EtCO_2_ signal, linearly interpolated, and resampled with each cardiac cycle. The instantaneous relationship between BP and CBFv was used to estimate critical closing pressure (CrCP) and resistance area product (RAP) for each cardiac cycle using the first harmonic method, as described in Panerai [15]. Beat-to-beat data were spline interpolated and resampled at 5 samples s^− 1^ to produce signals with a uniform time-base.

Dynamic cerebral autoregulation (CA) represents the transient relationship between BP (stimulus or input) and CBF (response or output), and was calculated by transfer function analysis (TFA), using the parameters gain, phase and coherence, and autoregulatory index (ARI), as previously described [16, 17]. Gain and phase reflect the relative amplitude and temporal relationship between the changes in BP and CBFv over a specified frequency range, respectively. Low gain indicates intact CA, whereas low phase (oscillations of CBFv and BP are synchronous) impaired CA. Coherence represents the fraction of output power that can be linearly explained by input power. Similarly to a correlation coefficient, it ranges from zero to 1. In the absence of a linear relationship, low signal-to-noise ratio, or other variables contributing to output changes, coherence will approach zero and will reduce the reliability of gain and phase estimates. These parameters were then averaged for the very-low frequency (VLF, 0.02–0.10 Hz), low frequency (LF, 0.10–0.25 Hz) and high frequency (0.25–0.40 Hz) ranges. Inverse FFT was then applied to the TFA, converting data back into the time domain, to calculate the CBFv step response. ARI was assigned to each recording by using the best least squares fit between the CBFv step response and one of the 10 model ARI curves proposed by Tiecks et al. [18]. Values of ARI = 0 represent absence of CA, while ARI = 9 corresponds to best-observed autoregulation. ARI was calculated for each participant for both hemispheres at baseline.

### Statistical analysis

Data were analysed using commercially available statistical software (SPSS 18.0, Chicago, IL). Shapiro-Wilk test was adopted to identify parameters that did not fit a normal distribution, which were then log transformed and tested again for normality. Inter-group differences were tested using χ^2^ test for categorical data and Student’s t-test for continuous variables. Within the stroke and control groups, intra-individual differences in CBFv, coherence, gain, phase and ARI, between LEI and SP centres were tested using two-way ANOVA with Tukey’s post hoc test comparisons performed when appropriate. In addition, Levene’s test was used to assess the equality of variances between the populations’ variances. Levels of *p* < 0.05 were considered statistically significant. The minimum sample size in each group was calculated by the formula described by Brodie et al. [19]: 44.6/∆ARI^2^ (where ∆ARI is the expected change in ARI) with 80% power at α = 0.05.

## Results

A total of 100 participants were recruited from both centres, corresponding to 25 AIS patients and 25 age- and sex-matched control subjects from each centre. Baseline demographic and clinical data are described in Tables 1 and 2, respectively. No significant differences were seen in baseline demographic data, though African ethnicity, dyslipidaemia, cardiomyopathy and atrial fibrillation were more frequent in SP, and sub-surgical carotid artery stenosis (< 50%) more frequent in the LEI stroke population (Table 1). Stroke patients received medication according to local guidelines for secondary prevention at the time of assessment, with the most common antihypertensive treatment including: beta-blockers (LEI = 14 (56%) /SP = 13 (52%)), angiotensin converting enzyme inhibitors (LEI = 11 (44%)/SP = 12 (48%)), diuretics (LEI = 10 (40%) /SP = 7 (28%)). Statin therapy was prescribed to three LEI and two SP patients.

**Table 1:** Baseline characteristics of the stroke and control participants by geographical population

Variables	Stroke		Controls	
	LEI (*n* = 25)	SP (*n* = 25)	LEI (*n* = 25)	SP (*n* = 25)
Age, years	60.6 (15.8)	62.4 (11.8)	61.1 (9.9)	60.7 (10.0)
aNIHSS	11.5 (6.6)	14.3 (5.9)	NA	NA
Sex, n (%)				
Female	10 (40)	12 (48)	10 (40)	11 (44)
Male	15 (60)	13 (52)	15 (60)	14 (56)
Ethnicity, n (%)				
Caucasian	20 (80)	15 (60)	23 (92)	22 (88)
African	4 (16)	9 (36)*	2 (8)	3 (12)
Natives	0	1(4)	0	0
Asian	1(4)	0	0	0
Diabetes, n (%)	2 (8)	4 (16)	0	0
Hypertension, n (%)	6 (24)	10 (40)	2 (8)	0
Dyslipidemia, n (%)	0	4 (16)*	0	0
ICA stenosis, n (%)	4 (16)*	0	0	0
HIV, n (%)	1 (4)	0	0	0
AF, n (%)	0	6 (24)*	0	0
Cardiomyopathy, n (%)	0	3 (12)*	0	0

Data are mean (standard deviation) or n (%)

*LEI* Leicester, *UK* SP, Sao Paulo, Brazil, *aNIHSS* admission National Institutes of Health Stroke Scale, *ICA* internal carotid artery, *HIV* human immunodeficiency virus, *AF* atrial fibrillation

*p < 0.02 for comparisons between LEI and SP

**Table 2:** Stroke characteristics by region

	LEI (*n* = 25)	SP (*n* = 25)
Stroke Side, n (%)		
Left	11 (44)	12 (48)
Right	14 (56)	13 (52)
Stroke aetiology, n (%)		
CE	14 (56)	18 (72)
LAA	3 (12)	0
SVD	7 (28)*	0
SOE	0	0
SUE	1 (4)	7 (28)*
Stroke type, n (%)		
PACS	14 (56)	15 (60)
TACS	4 (16)	10 (40)*
LACS	7 (28)*	0
Thrombolysis, n (%)	5 (20)	9 (36)

Data are presented as n (%)

*LEI* Leicester, *UK* SP, São Paulo, Brazil, *CE* cardiac embolism, *LAA* large-artery atherosclerosis, *SVD* small vessel disease, *SOE* stroke of other determined aetiology, *SUE* stroke of undetermined aetiology, *PACS* partial anterior circulation stroke, *TACS* total anterior circulation stroke, *LACS* lacunar circulation stroke

*p < 0.02 for comparisons between LEI and SP

Compared to LEI stroke patients, SP patients were more likely to have total anterior circulation stroke syndrome and stroke of undetermined aetiology, and less likely to have had a lacunar stroke and small-vessel disease aetiology, though there were no differences in thrombolysis rates (Table 2). No differences between time of stroke onset and haemodynamics assessment was found (LEI = 42.0(5.9) and SP = 39.3(7.6) hours).

After confirming a lack of significant inter-hemispheral difference, cerebral haemodynamic parameters (CBFv, CrCP, RAP, ARI, gain, phase and coherence) from the right and left hemispheres in control subjects were merged, as performed previously [13]. Table 3 gives the results of Levene’s tests for equality of variances between LEI and SP populations. The results indicate that the population variances across regions are equivalent.

**Table 3:** Levene’s test (statistics based on means) of variances equality between LEI and SP (geographical region) and stroke and control participants (participants type)

	Geographical Region		Participants Type	
	*F*	*p* value	*F*	*p* value
CBFv, cm.s^−1^	0.721	0.399	0.195	0.662
CrCP, mm Hg	4.276	0.080	3.153	0.091
RAP, mmHg.s.cm^− 1^	1.496	0.230	2.231	0.120
Coherence VLF range	2.099	0.493	2.443	0.407
Coherence LF range	1.727	0.610	1.987	0.540
Coherence HF range	0.929	0.703	1.592	0.622
Normalized gain VLF range, % mm Hg^− 1^	0.331	0.901	0.486	0.838
Normalized gain LF range, % mm Hg^− 1^	0.297	0.882	3.089	0.567
Normalized gain HF range, % mm Hg^−1^	3.921	0.098	3.444	0.612
Phase VLF range, radians	2.982	0.801	2.491	0.475
Phase LF range, radians	2.001	0.712	1.984	0.399
Phase HF range, radians	2.665	0.723	2.091	0.523
ARI	0.078	0.762	0.510	0.950

*CBFv* Cerebral blood flow velocity, *CrCP* Critical closing pressure, *RAP* Resistance Area Product, *VLF, LF, HF* Very low, low and high frequency ranges, respectively

Compared to control subjects, stroke patients had significantly higher mean arterial BP, and significantly reduced CBFv in the affected, but not unaffected, hemisphere at the time of assessment (Table 4). RAP was significantly higher in the affected hemisphere than both the unaffected hemisphere in stroke patients, and control subjects (Table 4). No significant geographical differences were seen in either systemic or cerebral haemodynamic parameters for both stroke patients and control subjects (Table 4).

**Table 4:** Systemic and cerebral hemodynamic parameters in stroke and control participants by region

Variables	Stroke Patients				Controls	
	SP (*n* = 25)		LEI (*n* = 25)		SP (*n* = 25)	LEI (*n* = 25)
	AH	UH	AH	UH		
MAP, mmHg	109.2 (5.7)^#^		103.0 (5.5)^#^		90.0 (6.1)	89.2 (3.9)
CBFv, cm.s^−1^	47.5 (6.3)^#^	55.1 (5.8)	41.7 (2.4)*	45.03 (2.8)	60.3(11.6)	53.8 (5.5)
CrCP, mm Hg	18.2 (6.7)	18.0 (4.6)	19.4 (5.0)	17.0 (5.5)	14.2 (4.47)	15.5 (8.0)
RAP, mmHg.s.cm^− 1^	1.97 (0.32)*^#^	1.76 (0.25)^#^	2.25 (0.23)*^#^	1.77 (0.17)^#^	1.56 (0.27)	1.65 (0.60)

Data are presented as mean (SD)

*LEI* Leicester, *UK* SP, Sao Paulo, Brazil, *AH* affected hemisphere, *UH* unaffected hemisphere, *MAP* Mean arterial pressure, *CBFv* Cerebral blood flow velocity, *CrCP* Critical closing pressure, *RAP* Resistance Area Product

*p < 0.01, Tukey’s post-hoc test for comparisons between stroke affected and unaffected hemispheres

#*p* < 0.05, Tukey’s post-hoc test for comparisons between controls and stroke participants (same geographical region)

With respect to CA parameters, compared to control subjects, coherence was significantly increased in both the affected and unaffected hemispheres of AIS patients for both LF and HF ranges (Table 5). In addition, both phase in the VLF range and ARI were significantly reduced in the affected and unaffected hemispheres of AIS compared to control subjects (Table 5). No significant regional differences were observed, except for gain in the HF range between the affected and unaffected hemisphere in SP patients, which was not found in LEI patients (Table 5). Univariate ANCOVA adjusted for stroke aetiology and subtypes of the cerebral haemodynamics parameters also did not revealed any differences between regions (Table 6).

**Table 5:** Dynamic CA parameters in stroke and control participants by geographical region

Variables	Stroke Patients				Controls	
	SP (*n* = 25)		LEI (*n* = 25)		SP (*n* = 25)	LEI (*n* = 25)
	AH	UH	AH	UH		
Coherence VLF range	0.65 (0.19)	0.64 (0.14)	0.61 (0.14)	0.59 (0.14)	0.52 (0.16)	0.48 (0.11)
Coherence LF range	0.68 (0.23)^#^	0.73 (0.19)^#^	0.68 (0.17)^#^	0.71 (0.15)^#^	0.56 (0.18)	0.58 (0.15)
Coherence HF range	0.72 (0.18)^#^	0.77 (0.10)^#^	0.69 (0.17)^#^	0.67 (0.16)^#^	0.52 (0.21)	0.51 (0.15)
Normalized gain VLF range, % mm Hg^− 1^	1.26 (0.57)	1.17 (0.66)	0.94 (0.33)	0.91 (0.34)	1.01 (0.39)	1.19 (0.43)
Normalized gain LF range, % mm Hg^− 1^	1.32 (0.48)	1.22 (0.46)	1.35 (0.44)	1.26 (0.69)	1.45 (0.83)	1.49 (0.78)
Normalized gain HF range, % mm Hg^− 1^	1.50 (0.74)*	1.36 (0.51)	1.36 (0.54)	1.28 (0.55)	1.35 (0.63)	1.40 (0.58)
Phase VLF range, radians	0.53 (0.42)^#^	0.78 (0.39)^#^	0.58 (0.50)^#^	0.70 (0.49)^#^	0.88 (0.35)	0.87 (0.41)
Phase LF range, radians	0.30 (0.45)	0.25 (0.38)	0.30 (0.35)	0.31 (0.39)	0.48 (0.19)	0.45 (0.41)
Phase HF range, radians	0.09 (0.29)	0.10 (0.25)	0.11 (0.40)^#^	0.17 (0.21)^#^	0.05 (0.21)	0.01 (0.28)
ARI	4.8 (2.3)^#^	4.9 (2.0)^#^	5.1 (1.8)^#^	5.0 (1.1)^#^	5.9 (1.5)	5.5 (1.2)

Data are presented as mean (SD)

*LEI* Leicester, *UK* SP, Sao Paulo, Brazil, *AH* affected hemisphere, *UH* unaffected hemisphere, *ARI* Autoregulatory Index, *VLF, LF, HF* Very low, low and high frequency ranges, respectively

*p < 0.01, Tukey’s post-hoc test for comparisons between stroke affected and unaffected hemispheres

#*p* < 0.05, Tukey’s post-hoc test for comparisons between controls and stroke participants

**Table 6:** ANCOVA and ANOVA results for comparison of cerebral haemodynamics parameters between geographical regions (stroke only)

	MS		F-statistic		*p*-Value	
	ANCOVA	ANOVA	ANCOVA	ANOVA	ANCOVA	ANOVA
CBFv, cm.s^−1^	3.2	8.6	0.7	0.9	0.83	0.42
CrCP, mm Hg	2.5	3.0	5.2	4.8	0.60	0.81
RAP, mmHg.s.cm^− 1^	4.9	3.0	6.1	1.8	0.10	0.24
Coherence VLF range	5.7	9.2	3.2	4.5	0.31	0.19
Coherence LF range	3.8	4.4	2.9	4.3	0.44	0.31
Coherence HF range	3.1	5.3	2.3	3.6	0.56	0.29
Normalized gain VLF range, % mm Hg^− 1^	14.0	5.1	5.2	1.4	0.07	0.33
Normalized gain LF range, % mm Hg^−1^	8.1	4.5	0.9	2.3	0.30	0.12
Normalized gain HF range, % mm Hg^−1^	7.8	4.4	5.5	1.2	0.21	0.40
Phase VLF range, radians	4.2	2.5	0.2	0.1	0.90	0.90
Phase LF range, radians	6.7	1.8	0.2	0.4	0.80	0.90
Phase HF range, radians	5.5	1.4	1.0	0.2	0.80	0.30
ARI	1.5	1.9	4.5	3.9	0.51	0.77

The analysis of covariance (ANCOVA) model included stroke aetiology and subtypes as covariates

*ANOVA* analysis of variance, *MS* mean square, *AH* affected hemisphere, *UH* unaffected hemisphere, *VLF, LF, HF* Very low, low and high frequency ranges, respectively

*p*-value for ANOVA (geographical interaction) and ANCOVA controlled for stroke aetiology and subtype

## Discussion

### Main findings

To the best of our knowledge, this is the first study to compare CA in participants from different geographical regions with marked environmental and socio-economic differences. These pooled analyses suggest no geographical differences in key, commonly measured cerebral haemodynamic parameters, including CBFv, CrCP, RAP and ARI. Overall, and in keeping with previous studies, impairment of cerebral haemodynamic parameters was reported in stroke compared to control participants, particularly in the affected hemisphere. Importantly, no geographical regional differences were found.

### Clinical relevance

Recognizing similarities and differences in the cerebral haemodynamics in healthy subjects and stroke has public health and clinical implications for several cardiovascular outcomes. Haemodynamic abnormalities may be useful early clinical markers in the development of age-related vascular neurodegenerative disorders. Describing cerebral haemodynamic changes and their regulation in different geographical populations may help understand differences across age and ethnicities, as well as the potential generalizability of clinical studies results.

Impairment of CBFv and CA has a direct and major impact on secondary brain injury and clinical outcomes [3, 4], as well as on planning effective therapeutic strategies that consider BP management and early mobilization protocols. In line with previous studies, our results showed a deterioration of important haemodynamic parameters in the acute phase of ischaemic stroke, particularly reduced phase and ARI values compared to controls [3, 20–23]. Moreover, no significant alteration in gain between groups was found [6, 20]. Though TFA became a popular approach for assessment of dynamic CA, very few acute stroke studies have reported the use of coherence estimates [3, 22]. These previous studies failed to find raised coherence values in acute stroke, but an analogous parameter (the squared correlation coefficient) was found to be increased bilaterally after the first 48 h of stroke onset, suggesting worsening of CA [23].

The importance and novelty of our results, however, stem from the similarities of the haemodynamic responses from research centres in both UK and Brazil, irrespective of the participant group (stroke or healthy controls). Despite the potential differences in dietary habits and lifestyles, and particularly in SP participants, higher prevalence of uncontrolled hypertension and widespread use of over-the-counter medications, cerebral haemodynamic parameters were not significantly different in our study. The authors believe that the outcome of this paper will strengthen the argument in favour of multicentre and multinational collaborative studies of the impact of cerebral haemodynamics in stroke and other conditions, such as sepsis and traumatic brain injury.

### Environment effects on cerebral haemodynamics

Since differences in CBF and its control mechanisms between European and South American populations have not been previously reported, it is difficult to assess consistency with other studies in the literature. A previous TCD study of healthy young participants (*n* = 20) in Germany and Hong Kong described no CBFv differences in the posterior cerebral arteries at rest and during cerebral activation [24]. Nevertheless, slower initial haemodynamic responses to visual activation paradigms were described in the Asian group that may be related to deficits in the nitric oxide system. Previous studies have also compared cerebral haemodynamic responses between South Asian and Caucasian participants, but the results were inconsistent. In a United Kingdom-based study, resting MCA CBFv and cerebrovascular resistance parameters (Pourcelot’s resistive index and Gosling’s pulsatility index) were significantly higher, and CA impaired in South Asian participants [25]. By contrast, no difference in the same parameters of CBFv and cerebrovascular resistance was found in another recent study of Canadian-based South Asians and Caucasians [26].

Disparities in cardiovascular and cerebrovascular health and mortality among populations have been well documented, but poorly understood [27, 28]. Studies have shown that the prevalence and mortality from hypertensive heart disease, stroke, and renal disease are higher among individuals of African compared to Caucasian descent [29]. Moreover, intracranial atherosclerosis is highly prevalent among patients with Asian, Hispanic, and African ancestry [30].

The present study did not demonstrate significant geographical differences in cerebral haemodynamic parameters between UK and Brazilian populations, with exception of gain at high frequency band. Immink and colleagues (2005) found an increase in gain only at higher frequencies in MCA-only stroke group. This may be the reason for the difference between regions, as SP comprised MCA infarcts only, whereas seven lacunar strokes were included in the LEI group (Table 2). Though recommended by the Cerebral Autoregulation Network (CARNet) [31], there is limited information in the literature on CA status in the complete frequency dependence of coherence, gain and phase in the range 0.02–0.40 Hz. While the TFA parameters changes in VLF and LF ranges are familiar, very little is known about the behaviour of the BP/CBFv system at frequencies higher than 0.25 Hz. In the TFA model, phase and gain are considered two different aspects of a high-pass filter that acts primarily in the VLF and LF ranges. At high frequencies, CA is considered less relevant and CBFv changes are associated with heart stroke volume beat-to-beat variability. More clinical studies are necessary to investigate the clinical importance of investigating CA in the higher frequency band, taking advantage of the work that has already been done. A further finding of the present study related to the responses of cerebrovascular resistance mechanisms, derived from a two-parameter model: RAP-CrCP. Levene’s test revealed a marginal difference in CrCP between geographical regions, with a tendency to higher values in LEI participants. Further research is needed to assess the clinical value of this finding.

### Standardized protocol

The experimental design of previous cerebral haemodynamic studies has been inconsistent, making it difficult to compare results directly [32, 33]. Furthermore, there have been no previous CA studies in either older or stroke populations in Brazil. In contrast, in the UK, some studies have previously described the haemodynamic responses to spontaneous BP fluctuations, carbon dioxide modulations and brain activation paradigms in both populations [12, 13, 34, 35]. More recently, the Department of Cardiovascular Sciences at the University of Leicester (Leicester, UK) has constructed a large database incorporating recordings from a series of separate studies performed in the same laboratory, using similar protocols, operator training and equipment [36]. They have presented normative values for cerebral haemodynamic parameters in a large healthy population indicating parameters that may help distinguish between normal and abnormal CA.

The present study was only possible due to the development of standardized data collection and analysis that provided a robust approach for the systematic evaluation of CBFv and its main regulatory mechanisms [37]. Similar to the Leicester normative study [36], all data collected in this study were acquired with similar study protocols and laboratory set-up, albeit with minor differences in the equipment used, and most importantly, without observer variability since all recordings were performed by only one researcher (AS). This avoids inter-observer variability in the study protocol (particularly concerning the TCD data), and it consequently increases the reproducibility of study reports.

### Study limitations

This study has limitations, including the use of non-invasive measurements of CBF and BP. Another limitation to consider is stroke patients received medication according to local guidelines for secondary prevention at the time of assessment. Though patients were on vasoactive therapy at time of assessment, no significant difference between populations was found. Although TFA and the ARI index can be regarded as the most widely used approach for assessment of dynamic CA, it is important to note that neither can be regarded as a ‘gold standard’. Future studies are needed to replicate our findings using alternative approaches such as time-domain analysis or the Mx index [12]. Finally, the sample size is relatively small, and is unlikely to be representative enough to ensure robust conclusions. Therefore, the authors strongly advocate a large multicentre validation study with larger sample size to explore further the possibility of regional geographical influences on cerebral haemodynamics, and possible mechanisms to support any differences.

## Conclusion

In conclusion, our results showed no significant differences in selected cerebral haemodynamic parameters between two different socio-economical geographical regions. In both research centres, acute ischaemic stroke depressed key measures of CA compared to a healthy older control population. These findings encourage further larger international studies of cerebral haemodynamic changes following AIS by pooling individual subject’s data from different regions.

## Abbreviations

AIS: Acute ischaemic stroke
ARI: Autoregulatory index
BP: Blood pressure
CA: Cerebral autoregulation
CBF: Cerebral blood flow
CBFv: Cerebral blood flow velocity
CrCP: Critical closing pressure
CT: Computerized tomography
EtCO_2_: End-tidal CO_2_
HR: Heart rate
LEI: Leicester
MCA: Middle cerebral artery
MRI: Magnetic resonance image
NHISS: NIH Stroke Scale
RAP: Resistance area product
SP: Sao Paulo
TCD: Transcranial doppler ultrasound
TFA: Transfer function analysis
